# Improving a Mg/S Battery with YCl_3_ Additive and Magnesium Polysulfide

**DOI:** 10.1002/advs.201800981

**Published:** 2018-12-12

**Authors:** Yan Xu, Guangmin Zhou, Shuyang Zhao, Wanfei Li, Feifei Shi, Jia Li, Jun Feng, Yuxing Zhao, Yang Wu, Jinghua Guo, Yi Cui, Yuegang Zhang

**Affiliations:** ^1^ School of Nano‐Tech and Nano‐Bionics University of Science and Technology of China Hefei Anhui 230026 China; ^2^ i‐lab Suzhou Institute of Nano‐Tech and Nano‐Bionics Chinese Academy of Science Suzhou Jiangsu 215123 China; ^3^ Department of Materials Science and Engineering Stanford University Stanford CA 94305 USA; ^4^ Laboratory for Computational Materials Engineering Shenzhen Tsinghua University Shenzhen Guangdong 518055 China; ^5^ School of Chemistry Biology and Material Engineering Suzhou University of Science and Technology Suzhou 215009 China; ^6^ Advanced Light Source Lawrence Berkeley National Laboratory Berkeley CA 94720 USA; ^7^ Department of Physics Tsinghua University Beijing 100084 China; ^8^ Stanford Institute for Materials and Energy Sciences SLAC National Accelerator Laboratory 2575 Sand Hill Road Menlo Park CA 94025 USA

**Keywords:** electrolyte additives, magnesium polysulfide cathodes, magnesium/sulfur batteries

## Abstract

Rechargeable magnesium/sulfur (Mg/S) batteries are widely regarded as one of the alternatives to lithium‐ion batteries. However, a key factor restricting their application is the lack of suitable electrolyte. Herein, an electrolyte additive that can reduce the polarization voltage is developed and 98.7% coulombic efficiency is realized. The as‐prepared Mg‐ion electrolyte exhibits excellent Mg plating/stripping performance with a low overpotential of 0.11 V for plating process, and high anodic stability up to 3.0 V (vs Mg/Mg^2+^). When it is coupled with magnesium polysulfide, which has high reactivity and is homogeneously distributed on carbon matrix, the Mg/S cells deliver a good cycling stability with a high discharge capacity over 1000 mAh g^−1^ for more than 50 cycles.

Among all the energy storage devices, rechargeable lithium‐ion batteries (LIBs) represent the most advanced technology and dominate the commercial market. However, there exists concern on the limited availability of resources such as Co, Ni, and Li to meet the tremendous demand in the electric vehicles and grid energy storage. Therefore, exploring battery chemistries with abundant resources and low cost is necessary. With regard to these demands, one ideal cathode material is abundant, lightweight element sulfur which has been extensively investigated in lithium/sulfur battery with a theoretical energy density of 2500 Wh Kg^−1^.^1^ Nevertheless, limitation of lithium resources and safety concerns in dendritic Li growth are important incentives for the development of battery chemistries beyond lithium.^2^ Magnesium is a promising alternative to lithium metal anode.^3^ Unlike metallic lithium,^4^ Mg can be safely used as a metal anode because of its dendrite‐free plating and stripping processes.^5^ Furthermore, Mg is an earth abundant and more chemically inert element than Li with a high theoretical volumetric capacity of 3832 mAh cm^−3^.^6^, ^7^ When magnesium is coupled with a sulfur cathode, magnesium/sulfur (Mg/S) batteries have attracted great attention because of their great potential to deliver two to three times the energy density than that of the state‐of‐the‐art LIBs.^7^, ^8^ Despite these advantages, Mg/S battery is still in its juvenile stage owing to lack of suitable electrolyte as seen from Table S1 (Supporting Information). magnesium chloride‐alimnium chloride (MgCl_2_‐AlCl_3_) electrolyte system is a promising electrolyte system for Mg/S battery. Unlike bis(trifluoromethane)sulfonimide magnesium (Mg(TFSI)_2_), hexafluorophosphate magnesium (Mg(PF6)_2_), borofluoride magnesium (Mg(BF4)_2_), etc., MgCl_2_‐AlCl_3_ inorganic salt will not decompose on the surface of Mg anode and affect the Mg ion plating/stripping behavior. However, with a higher standard reduction potential (Al (vs SHE) = −1.66 V) compared with Mg, Al will codeposit with Mg, making a low columbic efficiency of magnesium anode. After a carefully screening, yttrium chloride (YCl_3_) is selected here as a potential candidate of electrolyte additive. This is because that Y ion has a low standard electrode potential (Y (vs SHE) = −2.372 V), preventing yttrium from precipitation during the magnesium plating process. Moreover, YCl_3_ is very reactive with water,^9^ and can serve as a “getter” material to eliminate trace water in the electrolyte. In addition, the introduction of MgCl_2_‐YCl_3_ as an inorganic electrolyte can avoid the decomposition of magnesium salt.^10^

YCl_3_ is drastically different from AlCl_3_ used as a common additive in previous studies. In most electrolyte systems of Mg/S battery with AlCl_3_, the cycling life is poor and the capacity decay is fast from the second cycle.^11^, ^12^, ^13^ Furthermore, the high standard reduction potential (Al (vs SHE) = −1.66 V) AlCl_3_ compared with Mg, necessitate an electrolytic conditioning process for efficient Mg electrodeposition.^14^ However, during the conditioning process, the irreversibly codeposition of aluminum with Mg also makes AlCl_3_ an unsatisfactory additive.^15^

Another factor that results in a high polarization voltage and rapid capacity decay in Mg/S batteries is the solid sulfur cathode, which always suffers from uneven distribution of S and high polarization.^12^, ^13^, ^17^ Unfortunately, all previously reported cathode materials in Mg/S cells are commercial solid sulfur.^11^, ^12^, ^13^, ^16^ Therefore, we propose to use magnesium polysulfide as a catholyte in place of traditional sulfur in order to achieve Mg/S batteries with better reversibility and cycle life. Herein, we develop a Mg/S battery enabled by the Y‐based inorganic salt electrolyte and the Mg polysulfide (MgPS) cathode. The newly designed electrolyte salt shows high anodic stability (≈3.0 V) and excellent cycling performance for Mg deposition/dissolution. When coupled with magnesium polysulfide cathode, the greatly improved performance of Mg/S battery with a high discharge capacity over 1000 mAh g^−1^ and a long cycle‐life for more than 50 cycles is demonstrated for the first time.

The synthetic route for the Y‐based electrolyte is shown in **Scheme** **1**. A stoichiometric reaction of MgCl_2_ (1 molar equiv.) with YCl_3_ (2 molar equiv.), was reacted at 120 °C in an ionic liquid, *n*‐methyl‐(*n*‐butyl) pyrrolidinium bis(trifluoromethanesulfonyl)imide (PYR_14_TFSI) (see experimental details in the Supporting Information). In the following step, equivalent volume of diglyme (DG) was added into the solution, and a transparent Y‐based electrolyte is obtained.

**Scheme 1:**
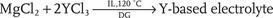
Synthetic route to newly designed Y‐based electrolyte.

To verify that yttrium is electrochemically stable during the Mg deposition process in the Y‐based electrolyte, the composition of deposited Mg is investigated by energy‐dispersive spectroscopy (EDS). A strong peak of Mg is shown in **Figure** **1**a. The weak peak of oxygen comes from a trace contamination due to a short exposure of the sample in air. The EDS data confirm that the deposit on the Mg electrode does not contain any yttrium. The electrochemical oxidative stability in Y‐based electrolyte is investigated by the linear sweep voltammetry. As illustrated in Figure 1b, the Y‐based electrolyte exhibits a high oxidative stability up to 3.0 V versus Mg/Mg^2+^ on Pt. In the gavalnostatic test, a symmetric Mg/Mg cell is cycled at 2 h charging and discharging intervals under a current density of 0.5 mA cm^−2^ (Figure 1c). It keeps a low polarization voltage at about 0.11 V (Figure 1c inset) and high reversibility over hundreds of plating/stripping cycles. The fluctuation at the first several cycles may come from the impurity of ionic liquid.^11^, ^13^ X‐ray photoelectron spectroscopy (XPS) was further employed to investigate the components of the solid electrolyte interphase (SEI) layer generated on the Mg metal surface. At the fluctuation stage, the SEI layer contains carbonyl group (288.0 eV, 290.2 eV (CO_3_)) and polyether carbon (286.0 eV (CH_2_O)) (Figure S1, Supporting Information). The surface species were also confirmed by O 1s spectra where the carbonyl (531.0 eV (C=O)), ether oxygen (533.0 eV (C—O—C)) was observed in O 1s spectra (Figure S1, Supporting Information), which indicate that organo‐Mg compound and magnesium carbonate (MgCO_3_) are the main components at the fluctuation stage. While after conditioning, the SEI layer contains magnesium oxide (MgO) (530 eV) only (Figure S1, Supporting Information). Oxide layer (specific resistance: 1 mΩ cm to 1 GΩ cm) usually has a better conductivity than carbonate and organo layers (specific resistance: 0.1–0.1 τΩ cm), so carbonate and organo magnesium components in the SEI layer result in the high polarization voltage and fluctuation in the initial stage.

**Figure 1:**
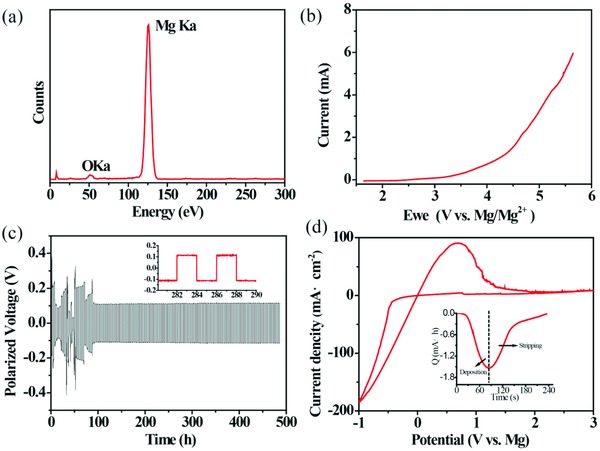
a) EDS of the deposited Mg on Pt electrode under a current density of 0.5 mA cm^−2^. b) Linear sweep voltammetry of the Y‐based electrolyte (IL:DG = 1:1). The working electrode is Pt while the counter and reference electrodes are Mg metal. Measurements are obtained at 25 mV s^−1^ under ambient conditions. c) Cycling behavior of a symmetrical cell with the Y‐based electrolyte (IL:DG = 1:1) at a current density of 0.5 mA cm^−2^. d) Cyclic voltammogram of the Y‐based electrolyte at 25 mV s^−1^. The working electrode is Pt while the counter and reference electrodes are Mg metals. The inset shows the plot of charge over time of Mg deposition and stripping.

To study the electrochemical Mg plating/stripping behavior in the as‐prepared electrolyte, cyclic voltammetry (CV) measurements were performed at a scan rate of 25 mV s^−1^ (Figure 1d). The plot of transferred charge amount over time is shown in the inset of Figure 1d, where the equivalent charges of deposition and stripping processes give a Coulombic efficiency (CE) as high as 98.7%, indicating that the as‐synthesized electrolyte enables reversible Mg deposition/dissolution. The cycling behavior of a Mg (5 mAh cm^−2^)|| Mg (100 mAh cm^−2^) cell was measured under 0.5 mA cm^−2^ (Figure S2, Supporting Information), where the polarization voltage did not show a dramatic increase within 146 cycles, illustrating an average CE higher than ≈97.3%. In contrast, in the Al‐based electrolyte, Al was codeposited with Mg during the process of Mg deposition (Figure S3a, Supporting Information). The oxidative stability of Al‐based electrolyte is narrow (2.8 V vs Mg/Mg^2+^) on Pt (Figure S3b, Supporting Information), and the polarization voltage is as high as 1.5 V at a current density of 0.5 mA cm^−2^ (Figure S3c, Supporting Information), which is not competitive compared with the Y‐based electrolyte.

**Scheme** **2** shows the synthetic process of MgS_8_ catholyte. Mg powder (1 equiv.) and S powder (8 equiv.) are reacted in a basic solvent of N‐methylimidazole (N‐MeIm) at 95 °C overnight.^18^ A reddish solution (Figure S4, Supporting Information) of MgS_8_ is resulted. The MgS_8_ is then attached onto a graphene/carbon nanotube (G‐CNT) matrix to form the magnesium polysulfide (MgPS) composite cathode. The details are described in the Experimental Section in the Supporting Information.

**Scheme 2:**
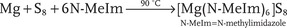
Synthetic route to MgS_8_.

**Figure** **2**a shows the configuration of Mg/S cell in which MgPS@G‐CNT and Mg foils are used as working and counter electrodes, respectively. The MgPS cathode was characterized with X‐ray absorption spectroscopy (XAS), ultraviolet–visible (UV–vis), X‐ray photoelectron spectroscopy (XPS), and scanning electron microscopy (SEM). As shown in Figure 2b, the S K‐edge XAS spectrum of the MgPS powder shows a shoulder peak at 2470 eV, which can be ascribed to the negatively charged terminal sulfur atoms in polysulfide chain. The peak at 2472 eV is from the neutral sulfur atoms inside the polysulfide chain.^18^ The XPS survey of a fresh MgPS cathode shows a Mg 2s peak at 89.5 eV and a S 2p_3/2_ peak at 164 eV (Figure 2c). As shown in Figure S5 (Supporting Information), the Mg 2s peak positioned at 89.5 eV can be ascribed to Mg^2+^ from MgPS. In the UV–vis absorption spectra of Mg polysulfide solution in dimethyl sulfoxide (DMSO) (Figure S6, Supporting Information), the peak at 495 nm can be assigned to S_8_^2−^, however, the peaks of S_6_^2−^ (358 nm) and S_3_^·−^ (616 nm) show up, due to partly disproportioned reaction occurs in Mg polysulfide solution.^19^ In Figure 2d, the S 2p3/2 XPS peaks positioned at 161.5, 163.5, and 165 eV correspond to the Mg—S bond in MgS, Mg—S bond in MgS*_x_*, and S—S bond in MgS*_x_*, respectively, and indicate the existence of polysulfide anions. The XPS results validate MgS*_x_* as the major component in the polysulfide electrolyte and are consistent with the XAS data and UV–vis data. The presence of MgS results from the disproportionation reactions of MgPS. Figure 2e,f shows the SEM images of G‐CNT and MgPS‐loaded G‐CNT. It can be seen that the MgPS is uniformly distributed on the surface of G‐CNT as confirmed by the corresponding EDS elemental mapping results (Figure S7, Supporting Information).

**Figure 2:**
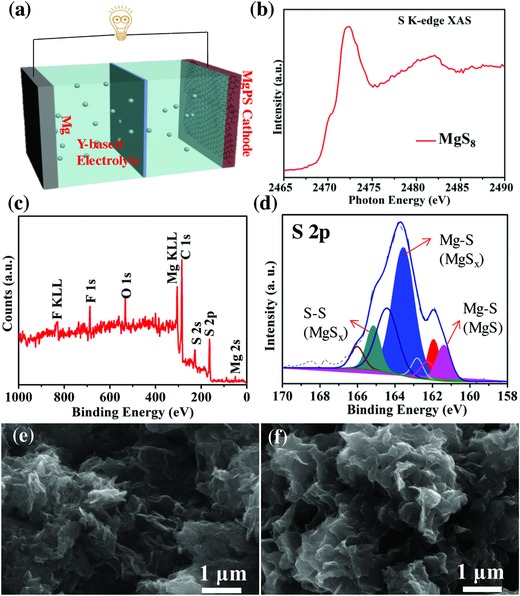
a) Schematic configuration of Mg/MgPS cell with the Y‐based electrolyte. b) The S K‐edge XAS of the MgPS powder. c) XPS survey curve of the MgPS@G‐CNT composite cathode material. d) S 2p XPS spectrum of the MgPS@G‐CNT. e,f) The SEM images of G‐CNT and MgS_8_@G‐CNT.

In order to examine the different effects of YCl_3_ additive and AlCl_3_ additive, we compare the electrochemical performances of two different Mg/S cells: MgPS cathode in Al‐based electrolyte, and MgPS cathode in Y‐based electrolyte. **Figure** **3**a shows the cycling performance of two cells at the current density of 83 mA g^−1^. When the MgPS cathode is used with the conventional Al‐based electrolyte, the performance in the first 10 cycles is maintained at 530 mAh g^−1^; however, its capacity starts to decay very quickly in the following cycles. Remarkably, when the Y‐based electrolyte is introduced, the capacity is greatly improved to more than 1000 mAh g^−1^ and the cell can be cycled for 50 cycles, in a sharp contrast with the previous case. It should be noted that the slightly lower capacity in the first few cycles can be ascribed to the activation process of MgPS in the Y‐based electrolyte. The typical voltage profiles of the cells with and without Y‐based electrolyte are compared in Figure 3b. With Y‐based electrolyte, the discharge/charge profiles show flat plateaus at ≈1.2 and ≈2.2 V, respectively. This is in contrast with other cells, where clear discharge plateaus could not be identified and the charging polarization voltages are as high as 2.4 V. We attribute the lower polarization voltage of the MgPS/Y‐based electrolyte cell to the better Mg plating/stripping behavior in YCl_3_‐based electrolyte and the homogeneously distribution of MgPS on the G‐CNT matrix. Electrochemical impedance spectroscopy (EIS) is performed for the two different Mg/S cells in order to compare their electrochemical kinetics. As shown in Figure 3c,d, the low impedance of the cell with Y‐based electrolyte verifies the synergetic effect of the newly developed Y‐based electrolyte additive and the MgPS cathode.

**Figure 3:**
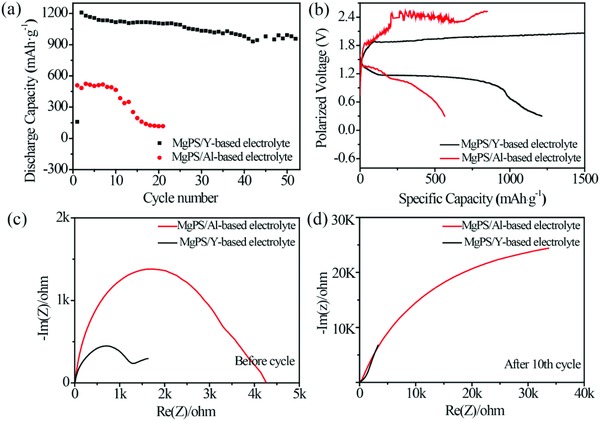
a) Cycling stability of the MgPS/Y‐based electrolyte, and MgPS/Al‐based electrolyte cells under a current density of 80 mA g^−1^. b) Discharge and charge profiles of the two different cells. c,d) EIS of two different cells before cycle and after ten cycles.

To understand the different performances of these two cells, we performed post‐analyses in SEM. The SEM images of the different cathodes loaded on carbon nanofiber (CNF) at the discharged and charged states are shown in **Figure** **4**. With Y‐based electrolyte, the surface of the MgPS@CNF cathode contains irregular‐shaped small particles at discharge states, which disappeared at charged state (Figure 4a,b). There is no obvious particle aggregation during cycling, and the CNF skeleton structure is well maintained. However, significant surface changes of the MgPS@CNF cathode in the conventional Al‐based electrolyte can be observed by comparing Figure 4c and 4d, where a large amount of aggregated particles can be observed on the surface. Furthermore, these large particles do not dissolve at the charge state. From SEM images, it can be concluded that the YCl_3_ additive and MgPS cathode can promote the decomposition of aggregated MgS particles, which results in a reduced polarization voltage and a significantly increased sulfur utilization. To investigate the different effects between the YCl_3_ and AlCl_3_ additives on the decomposition process of MgS, density functional theory (DFT) calculations were performed to derive the energy profiles of reactions. In the charge process, two Cl will transfer from YCl_3_ (or AlCl_3_) additives to Mg on the surface of MgS, resulting in the decomposition of MgS and formation of MgCl_2_. The transferring processes of Cl can be divided into two steps and expressed in the following reactions

(1) $${\mathrm{YCl}}_{3}+{\mathrm{Mg}}^{2+}\to {\mathrm{YCl}}_{2}{}^{+}+{\mathrm{MgCl}}^{+}\to {\mathrm{YCl}}^{2+}+{\mathrm{MgCl}}_{2}$$

(2) $${\mathrm{AlCl}}_{3}+{\mathrm{Mg}}^{2+}\to {\mathrm{AlCl}}_{2}{}^{+}+{\mathrm{MgCl}}^{+}\to {\mathrm{AlCl}}^{2+}+{\mathrm{MgCl}}_{2}$$

**Figure 4:**
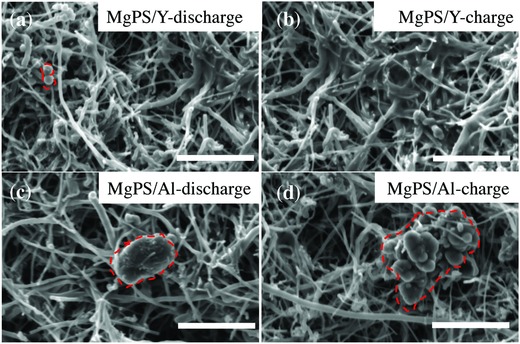
a,b) SEM images of the MgPS cathode discharged to 0.3 V and fully charged in the Y‐based electrolyte; c,d) the MgPS cathode discharged to 0.3 V and fully charged in the Al‐based electrolyte. Scale bar: 4 µm.

We took the (001) surface of MgS in NaCl‐type structure as the model to investigate the transferring processes of Cl from the YCl_3_ (or AlCl_3_) to the surface Mg. Figure S8 (Supporting Information) shows the adsorption conformations of YCl_3−_ *_x_* + MgCl*_x_* and AlCl_3−_ *_x_* + MgCl*_x_* (*x* = 0, 1, and 2) on MgS (001) surface. It shows that the adsorption conformation of YCl_3−_ *_x_* is similar to that of AlCl_3−_ *_x_*. Figure S9 (Supporting Information) schematically illustrates the energy profiles of transferring processes of Cl from YCl_3_ and AlCl_3_ to the surface Mg on MgS (001) surface. The first Cl transferring from YCl_3_ (or AlCl_3_) to the surface Mg with energy barrier of 1.80 eV (1.63 eV), followed by the second Cl from YCl_2_ (or AlCl_2_) to MgCl with barrier of 2.57 eV (2.94 eV). The higher energy barrier of the two steps determines the overall energy barrier of the decomposition of MgS and the formation of MgCl_2_. The overall energy barrier of YCl_3_ with 2.57 eV is less than that of AlCl_3_ by 0.37 eV, which indicates that YCl_3_ additive can improve the decomposition of MgS in comparison with AlCl_3_ additive.

In conclusion, a new type of inorganic Y‐based electrolyte with high plating/stripping coulombic efficiency is developed for Mg/S batteries. The newly designed electrolyte exhibits superior properties, including a good anodic stability (3.0 V vs Mg/Mg^2+^), an ultrahigh Mg plating/striping Coulombic efficiency (≈98.7%), and a long Mg plating/striping cycle life. When the Y‐based electrolyte is used together with the MgPS/G‐CNT cathode, the Mg/S cell demonstrates an ultrahigh capacity of 1000 mA h g^−1^ with an excellent cycling performance over 50 cycles. This new electrolyte additive offers new avenue for further development of better Mg electrolytes and practical Mg/S cells.

## Experimental Section

*Material Preparation*: Anhydrous magnesium chloride (MgCl_2_, 99.9%), aluminum chloride (AlCl_3_, 99.99%), and anhydrous DG were received from Sigma‐Aldrich, PYR_14_TFSI was purchased from MTI Corporation, and YCl_3_ was prepared by Prof. Y. M. Yao in Soochow University. All reactants and solvents, unless otherwise stated, were used as received. All the samples were handled in an argon‐filled glovebox with water below 0.5 ppm and oxygen below 15 ppm. MgCl_2_ (19 mg) and anhydrous YCl_3_ (78 mg) (or MgCl_2_ (19 mg) and anhydrous AlCl_3_ (53.3 mg)) were added to a 10 mL glass vial, which was then vigorously stirred at 120 °C in 1.5 mL PYR_14_TFSI overnight. Then 1.5 mL DG was added with stirring overnight at room temperature to form the Y‐based electrolyte. Treatment of 16.7 mg of Mg (0.688 mmol), 180 mg of sulfur (0.702 mmol), and 3 mL of N‐MeIm at 95 °C for 12 h to obtain a red solution. Then 360 mg G‐CNT was added and stirred overnight. 60 mg polyvinylidene fluoride (PVDF) dissolved in NMP was added to the above slurry. The resulting slurry was uniformly spread via a doctor blade on pyrolytic graphite. G‐CNT was pasted on pyrolytic graphite first and then MgPS solution was dropped onto G‐CNT. Then the electrode was dried at 60 °C in the glovebox. Typically, each electrode contains about 0.7–1.0 mg cm^−2^ of the active material.

*Materials Characterization*: The morphologies of the samples were investigated using an FEI XL30 Sirion SEM operated at an accelerating voltage of 5 kV. XPS analysis was performed on an SSI SProbe XPS spectrometer with monochromatic Al Kα (1486.6 eV) radiation.

*Electrochemical Measurements*: Electrochemical experiments were performed using CR2032 coin cells assembled in an argon‐filled glovebox with magnesium metal as the counter and reference electrodes and MgS_8_@G‐CNT as cathode. Galvanostatic cycling measurements were evaluated with a LAND battery test system. Cyclic voltammetry measurements were performed on a VMP3 potentiostat (Bio‐logic). EIS data were obtained on the same potentiostat from 200 kHz to 100 mHz with an AC voltage amplitude of 10 mV at the open‐circuit potential.

*Computational Details*: Spin‐polarization DFT calculations were performed by using Vienna Ab initio Simulation Package (VASP).^20^ The ion–electron interaction was described by the projector‐augmented wave (PAW) and exchange‐correlation interaction was described by Perdew–Burke–Ernzerhof functional (PBE) of generalized gradient approximation (GGA).^21^, ^22^ An energy cutoff of 400 eV was used for the plane‐wave basis set. The structure was fully relaxed until the forces were less than 0.02 eV Å^−1^. MgS(001) surface was modeled by a five‐layer slab with 4 × 4 supercell and only Γ point was used. The bottom two atomic layers of the slab model were fixed during structural optimizaiton. To eliminate the interaction between periodic slabs, the vacuum layer was set to 20 Å. DFT‐D3 method was used to describe van der Waals force.^23^ The climbing‐image nudged elastic band (CI‐NEB) method was used to determine the activation barriers and minimum energy paths.^24^

## Conflict of Interest

The authors declare no conflict of interest.

## Supporting information

Supplementary
