# Improving a Mg/S Battery with YCl_3_ Additive and Magnesium Polysulfide

**DOI:** 10.1002/advs.201903603

**Published:** 2020-01-22

**Authors:** Yan Xu, Guangmin Zhou, Shuyang Zhao, Wanfei Li, Feifei Shi, Jia Li, Jun Feng, Yuxing Zhao, Yang Wu, Jinghua Guo, Yi Cui, Yuegang Zhang

*Adv. Sci.* **2019**, *6*, 1800981

In the originally published version of this article, the SEM image in **Figure** **4**B inadvertently features the wrong sample. The correct Figure 4B is shown below. The authors declare that this correction does not affect the description, interpretation, or the original conclusions of the manuscript. The authors regret the inconvenience this error may have caused.

**Figure 4:**
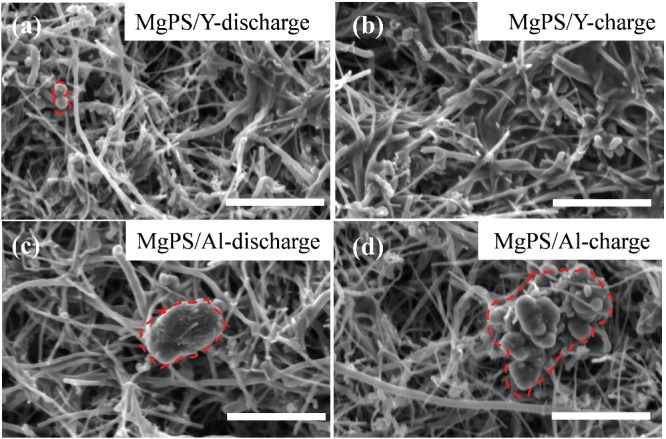
a,b) SEM images of the MgPS cathode discharged to 0.3 V and fully charged in the Y‐based electrolyte; c,d) the MgPS cathode discharged to 0.3 V and fully charged in the Al‐based electrolyte. Scale bar: 4 µm.

