# Supplementary material for: Improving a Mg/S Battery with YCl3 Additive and Magnesium Polysulfide
Source: Adv Sci (Weinh). 2018 Dec 12;6(4):1800981. doi: 10.1002/advs.201800981 (PMC6382296; doi:10.1002/advs.201800981)
Supplement: Supplementary file 1 — Supplementary [file ADVS-6-1800981-s001.pdf]

Copyright WILEY-VCH Verlag GmbH & Co. KGaA, 69469 Weinheim, Germany, 2018.

ADVANCED SCIENCE
Open Access

Supporting Information

for *Adv. Sci.,* DOI: 10.1002/advs.201800981

Improving a Mg/S Battery with YCl_3 Additive and Magnesium Polysulfide

Yan Xu, Guangmin Zhou, Shuyang Zhao, Wanfei Li, Feifei Shi, Jia Li, Jun Feng, Yuxing Zhao, Yang Wu, Jinghua Guo, Yi Cui,* and Yuegang Zhang**

WILEY-VCH

Copyright WILEY-VCH Verlag GmbH & Co. KGaA, 69469 Weinheim, Germany, 2018.

Supporting Information

Improving Mg/S battery by YCl_3 additive and magnesium polysulfide

Yan Xu, Guangmin Zhou, Shuyang Zhao, Wanfei Li, Feifei Shi, Jia Li, Jun Feng, Yuxing Zhao,

Yang Wu, Jinghua Guo, Yi Cui,* and Yuegang Zhang**

Table 1. Comparison of different electrolytes in defects, prepared method and compatibility with S aspects.

Electrolyte system	Defects	Prepared methodes	Compatability with S
[1]$MgCl_2$-$AlCl_3$	Al co-deposite with Mg	One-step	Yes
[2]$AlCl_3$-$Mg(HMDS)_2$	$HMDS^-$ decompose on Mg surface	Two-step	Yes
[3]$Mg(PF_6)_2$	Passivate Mg surface	One-step	Unknown
[4]$Mg(TFSI)_2$	Passivate Mg surface	One-step	Yes
[5]$Mg(BF_4)_2$	React with Mg	One-step	No
[6]$Mg(BH_4)_2$	Limited potential window	One-step	No
[7]$Mg(CB_{11}H_{12})_2$	Complicated synthetic procedure	complicated	Unknown
[8]$Mg(THFPB)_2$	Expensive	One-step	Yes
$MgCl_2$-YCl_3		One-step	Yes

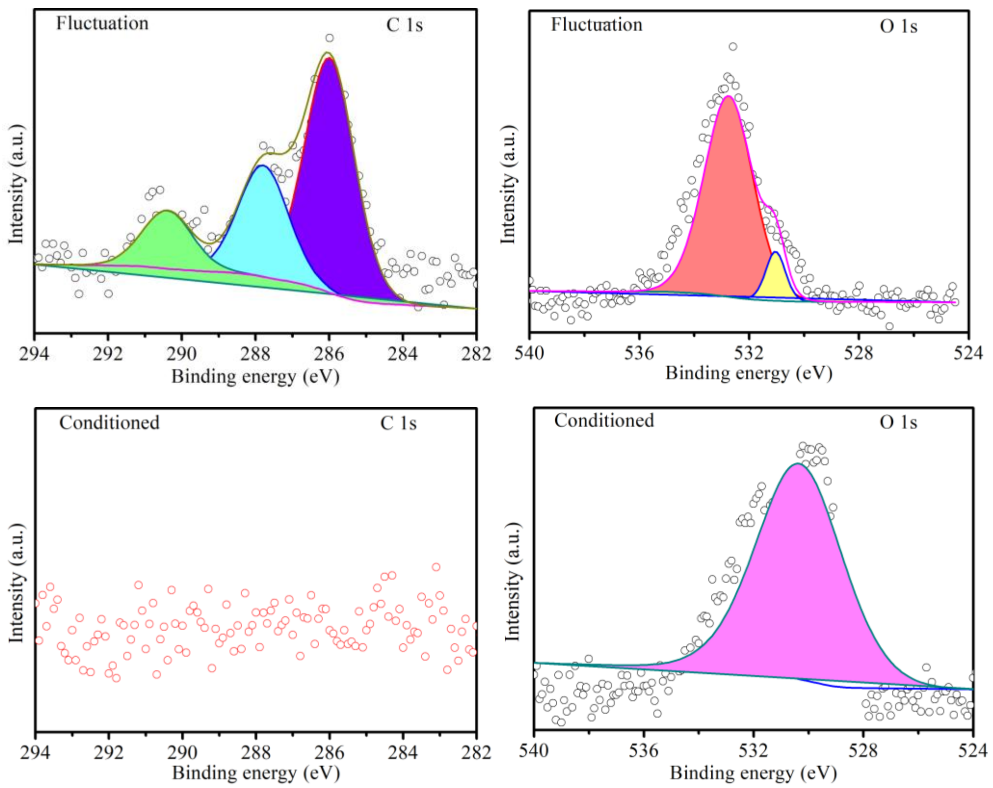

Figure S1. Characterize the components of SEI on cycled Mg metal at fluctuation stage and conditioned stage.

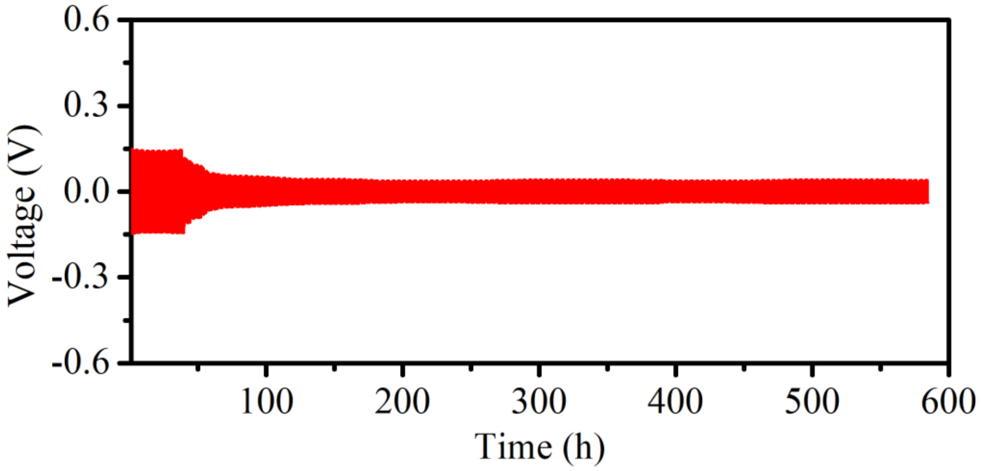

Figure S2. Electrochemical performance of Mg (5 mAh·cm^{-2}) ||Mg (100 mAh·cm^{-2}) cells for CE testing at current density of 0.5 mA·cm^{-2} and a capacity of 1 mAh cm^{-2}. The cycle time was 4 hours per cycle (2 hours charging and 2 hours discharging). **Average CE calculation methods：** In cycle life testing, the cell fails due to the depletion of electrolyte as a result of reaction with the deposited Mg metal. In this experiment, the cell cycled for 146 cycles without failure, which means there is at least 1 mAh·cm^{-2} equivalent amount of Mg remained on the Mg electrode in order to sustain the cycling to the capacity of 1 mAh·cm^{-2}. Because the Mg electrode (100 mAh·cm^{-2}) has excess Mg, the maximum equivalent amount of possible Mg consumption on the Mg electrode (5 mAh·cm^{-2}) is 4 mAh·cm^{-2} over 146 cycles. This corresponds to a maximum average capacity loss rate of 4 mAh·cm^{-2}/146 cycles/1 mAh·cm^{-2} = 2.7% per cycle. Therefore, the average Coulombic efficiency is ＞ 97.3% for the first 146 cycles.

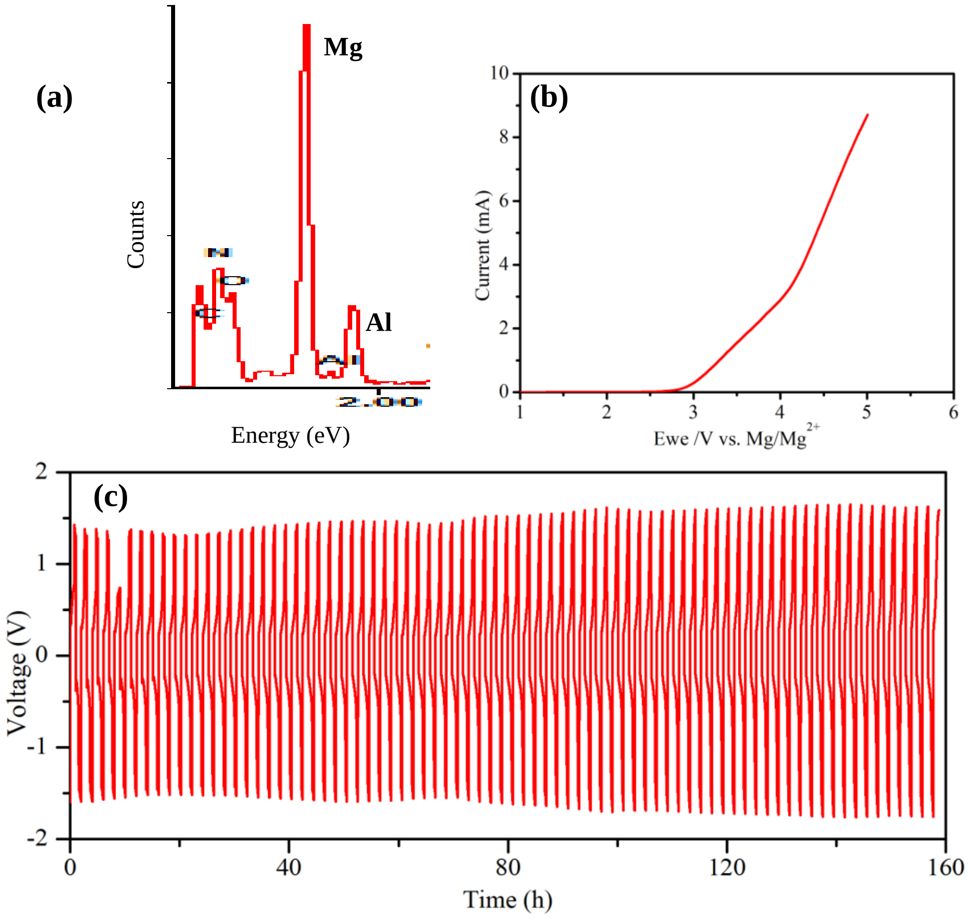

Figure S3. (a) EDS of the deposited Mg on Pt electrode under a current density of 0.5 mA cm^{-2} in the electrolyte of $MgCl_2$-$2AlCl_3$. (b) Linear sweep voltammetry of the Al-based electrolyte (IL:DG = 1:1). The working electrode is Pt while the counter and reference electrodes are Mg metal. Measurements are obtained at 25 mV·s^{-1} under ambient conditions. (c) Cycling behavior of a symmetrical cell with the Al-based electrolyte (IL:DG=1:1) at a current density of 0.5 mA cm^{-2}.

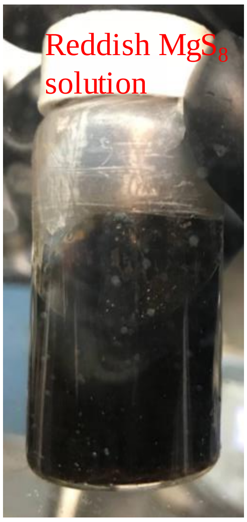

Figure S4. The image of the reddish MgS_8 solution.

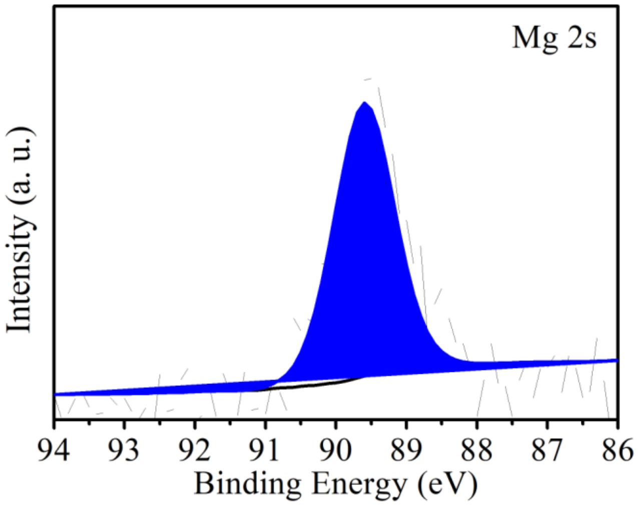

Figure S5. Mg 2s XPS spectrum of the MgS_8@G-CNT cathode.

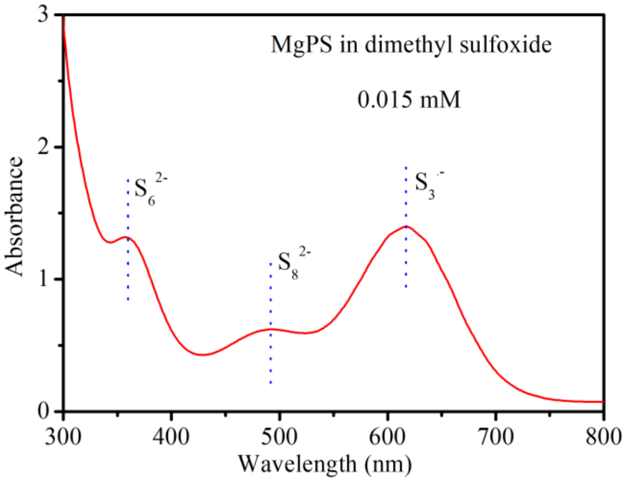

Figure S6. UV/Vis spectra of MgS_8 solutions in DMSO.

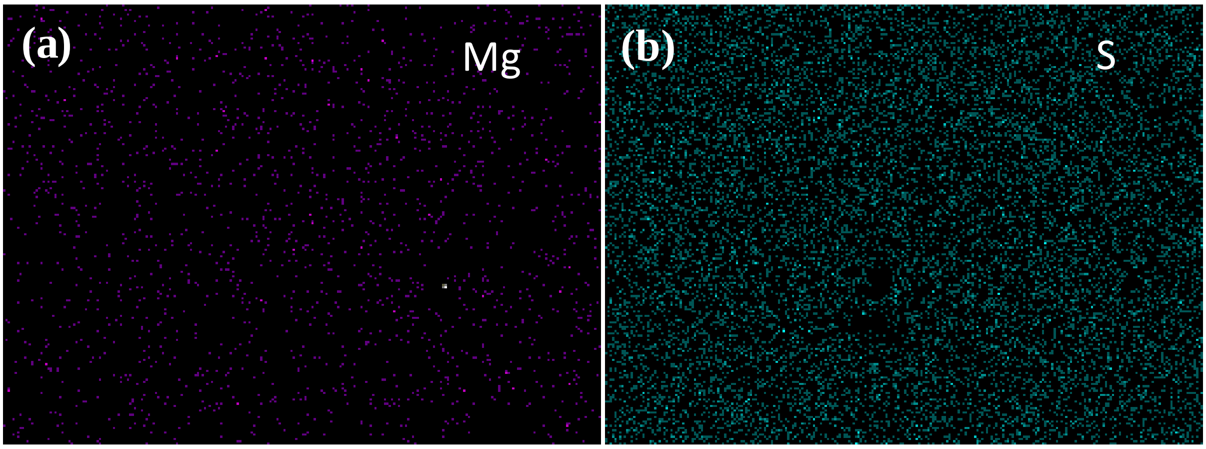

Figure S7. The EDS elemental mapping showing a uniform distribution of (a) Mg and (b) sulfur in the MgS_8@G-CNT cathode.

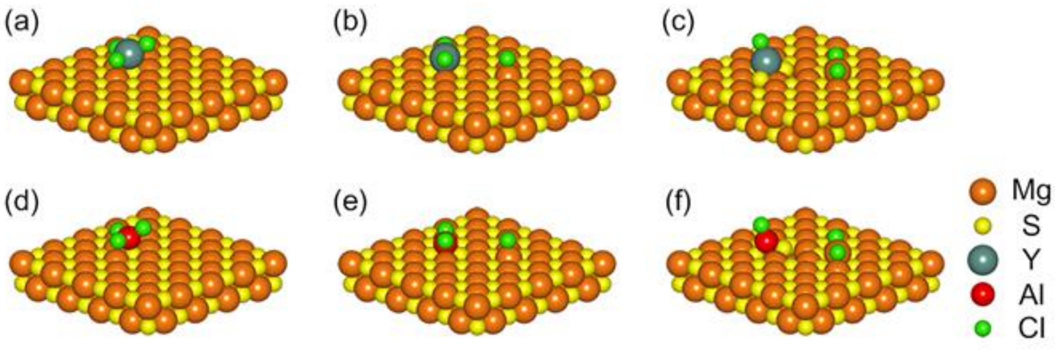

Figure S8. Adsorption conformations of (a) YCl_3+Mg, (b) YCl_2+MgCl, (c) YCl+$MgCl_2$, (d) $AlCl_3$+Mg, (e) $AlCl_2$+MgCl and (f) AlCl+$MgCl_2$ on the (001) surface of MgS in NaCl-type structure, respectively.

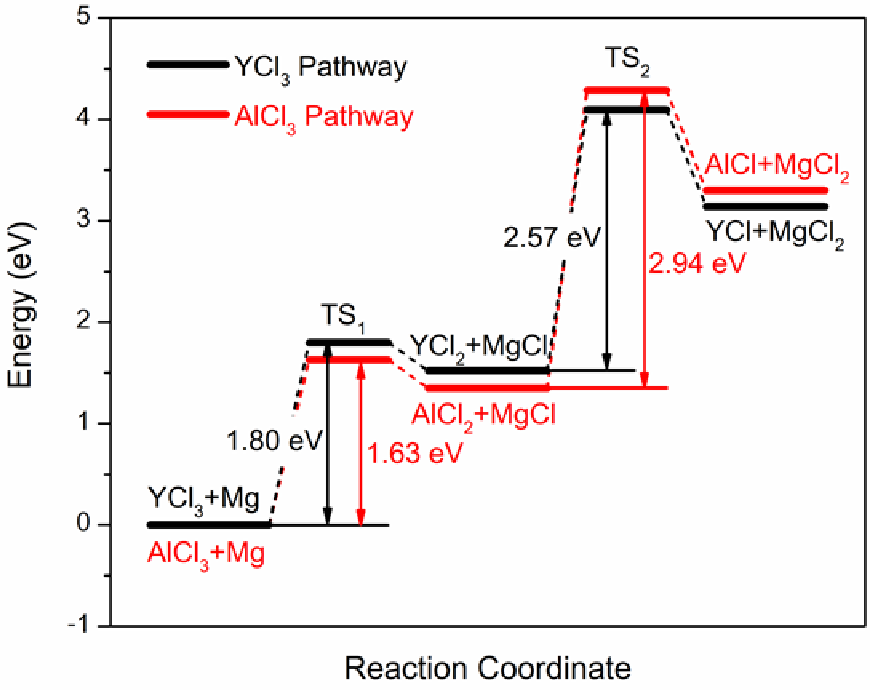

Figure S9. Energy profiles of transferring processes of Cl from YCl_3 (black) and $AlCl_3$ (red) to the surface Mg on MgS (001) surface, respectively.

References

(1) Li, W.; Cheng, S.; Wang, J.; Qiu, Y.; Zheng, Z.; Lin, H.; Nanda, S.; Ma, Q.; Xu, Y.; Ye, F.; Liu, M.; Zhou, L.; Zhang, Y. *Angewandte Chemie International Edition* **2016**, *55*, 6406.

(2) Xu, Y.; Li, W.; Zhou, G.; Pan, Z.; Zhang, Y. *Energy Storage Materials* **2018**, *14*, 253.

(3) Keyzer, E. N.; Glass, H. F. J.; Liu, Z.; Bayley, P. M.; Dutton, S. E.; Grey, C. P.; Wright, D. S. *Journal of the American Chemical Society* **2016**, *138*, 8682.

(4) Ha, S.-Y.; Lee, Y.-W.; Woo, S. W.; Koo, B.; Kim, J.-S.; Cho, J.; Lee, K. T.; Choi, N.-S. *ACS Applied Materials & Interfaces* **2014**, *6*, 4063.

(5) Vardar, G.; Sleightholme, A. E. S.; Naruse, J.; Hiramatsu, H.; Siegel, D. J.; Monroe, C. W. *ACS Applied Materials & Interfaces* **2014**, *6*, 18033.

(6) Kar, M.; Ma, Z.; Azofra, L. M.; Chen, K.; Forsyth, M.; MacFarlane, D. R. *Chemical Communications* **2016**, *52*, 4033.

(7) Tutusaus, O.; Mohtadi, R.; Arthur, T. S.; Mizuno, F.; Nelson, E. G.; Sevryugina, Y. V. *Angewandte Chemie International Edition* **2015**, *54*, 7900.

(8) Zhang, Z.; Cui, Z.; Qiao, L.; Guan, J.; Xu, H.; Wang, X.; Hu, P.; Du, H.; Li, S.; Zhou, X.; Dong, S.; Liu, Z.; Cui, G.; Chen, L. *Advanced Energy Materials* **2017**, *7*, 1602055.
